# Distribution and diversity of *Salmonella* serovars from beef cattle before, at, and after processing in relation to human-derived serovars

**DOI:** 10.3389/fvets.2026.1897484

**Published:** 2026-07-22

**Authors:** Emma Teague, Babafela Awosile

**Affiliations:** School of Veterinary Medicine, Texas Tech University, Amarillo, TX, United States

**Keywords:** cattle, human salmonellosis, multivariate analysis, One Health surveillance, *Salmonella enterica*

## Abstract

**Background:**

Globally, *Salmonella enterica* remains a leading cause of foodborne illness, and cattle serve as an important reservoir. Understanding serovar distribution across the beef production continuum and its contribution to human salmonellosis is critical for surveillance and control strategies.

**Methods:**

We analyzed *Salmonella* isolates from cattle (cecal, HACCP, retail meat), and human clinical sources using multivariate approaches, including principal component analysis (PCA), multidimensional scaling (MDS), analysis of similarities (ANOSIM), and permutational analysis of multivariate dispersions (PERMDISP).

**Results:**

Ordination analyses revealed clear ecological structuring of serovars by sampling source. *Typhimurium*, *Newport*, and *Enteritidis* dominated human isolates and exhibited the greatest diversity, consistent with multiple exposure pathways. Retail isolates formed a compact cluster that overlapped with human isolates and were associated with *Montevideo* and *Infantis*, suggesting that contaminated retail meat may contribute to human exposure. Cecal and HACCP isolates were the most homogeneous, clustering tightly with serovars such as *Cerro*, *Anatum*, *Kentucky*, *Mbandaka*, *Muenchen*, *Meleagridis*, and *Muenster*, reflecting stable pre-harvest reservoirs. ANOSIM confirmed significant ecological separation among sources (R = 0.512, *p* < 0.001), while PERMDISP revealed significantly greater variability among human isolates than among retail, HACCP, and cecal sources.

**Conclusion:**

*Salmonella* serovar distributions are shaped by sampling sources, with human and retail serovars aligning closely, cecal and HACCP serovars reflecting homogeneous cattle-associated pools, and human cases showing the broadest serovar diversity. These findings highlight retail meat as a critical transmission link, underscore the value and limitations of surveillance, and emphasize the need for integrated monitoring to improve understanding of *Salmonella* ecology across the One Health interface.

## 1 Introduction

*Salmonella* is a Gram-negative, rod-shaped bacterium and remains one of the most common causes of foodborne illness in the United States, causing over 400,000 AMR infections annually (1). With over 2,500 serovars, *Salmonella* can cause salmonellosis in people and many animal species worldwide (2, 3). These infections cause morbidity, mortality, and economic burden and are especially severe in infants, the elderly, or immunocompromised patients (4). An estimated 94% of *Salmonella* infections are caused by foodborne transmission (5). Nearly all types of foods can harbor *Salmonella*, as it is widely distributed in nature. There are multiple stages throughout food production where foods can become contaminated with *Salmonella* (6). For instance, a key moment of carcass and organ contamination can occur during the slaughtering of food animals (7). Fecal contamination can occur at many points throughout the slaughter and processing of beef cattle; therefore, proper preventive food safety measures must be taken. Feces from animals can also contaminate agricultural environments, leading to the possible contamination of plants and irrigation water (8). People with direct contact with these sources are at risk of *Salmonella* exposure that could lead to illness. Symptoms of salmonellosis can range from mild gastroenteritis to life- threatening, depending on the serovar that causes the infection (9). Some serovars are restricted to a single species, while others can infect multiple species (10, 11). The serotype involved in illness determines the severity of *Salmonella* infections in humans, which also depends on the host’s health status (7). People more susceptible to *Salmonella* infection include children under 5 years of age, the elderly, and patients with a weakened immune system, compared to healthy individuals (7). In all populations, failure to practice proper hygiene, such as handwashing or equipment and personal protective equipment (PPE) sanitation, can lead to *Salmonella* growth and exposure. This also applies to food hygiene practices, such as cleaning fruits and vegetables before use, adequate cooking of meats, pasteurization of dairy products and eggs, sanitation of the cooking environment, and proper food storage.

Foodborne transmission is regarded as the primary route of *Salmonella* infection, although some studies have shown risk factors for salmonellosis associated with direct contact with infected animals (5). The transmission of *Salmonella* from the foodborne route can occur through contamination of foods and water during slaughter, using manure as fertilizer, or irrigation water exposed to manure (5). Consequently, plants can also harbor pathogens and transmit *Salmonella* back to animals (8). The understanding that even low numbers of *Salmonella* can cause infection highlights the importance of strict food safety management (4). In mammals, *Salmonella* infection occurs after consumption of contaminated food or water (8). The most common food sources include undercooked eggs and meats, including poultry, beef, and pork, dairy products that have not been pasteurized, and raw vegetables (5). After ingestion of an infectious dose, the intestinal tract is colonized by the pathogen, thus causing sickness (12). Food animals, such as swine, poultry, and cattle, serve as a major source of *Salmonella* transmission and infection (7). Animal products are the main vehicle of *Salmonella* transmission due to *Salmonella’s* ability to survive in meats and animal products that are not cooked thoroughly (13). Direct exposure, including people interacting with dairy cattle in public settings, is also a risk for salmonellosis (5). This is due to fecal-oral contamination, environmental settings, and exposure to contaminated udders or raw milk. Due to the unpasteurized nature of raw milk, which acts as a reservoir for many different bacterial pathogens, including *Salmonella*, consumer ingestion can lead to *Salmonella* infection. Since milk is packed with nutrients, bacterial pathogens can grow and thrive in the ideal environment of raw milk, unless it is pasteurized. Ready-to-eat meals also pose a risk of *Salmonella* exposure to humans. Due to their heating requirements being minimal, improper food handling and storage, or undercooked ingredients, ready-to-eat meals can allow for the presence of *Salmonella* exposure. Direct contact with animals and other sources contaminated by animals is also a risk for infection.

Due to how common *Salmonella* infections are, integrated surveillance and monitoring programs have been supported by the World Health Organization (WHO) (14). Today, the United States Centers for Disease Control and Prevention (CDC) uses passive surveillance to collect data from confirmed *Salmonella* cases in the United States. The CDC calls this the Laboratory-based Enteric Disease Surveillance System (LEDS). The CDC also offers resources to inform the public of current and past *Salmonella* outbreaks, which are intended to educate people and inform them about active recalls. The National Antimicrobial Resistance Monitoring System (NARMS) is used in the United States to provide data for secondary use from positive *Salmonella* cases associated with humans, cattle, chickens, turkeys, and swine. The secondary bovine data provides samples taken from the cecal, hazard analysis and critical control point (HACCP), and retail aspects of meat from beef cattle, which represent different stages of potential pathogen contamination within food processing. The CDC also provides the public with access to pathogen surveillance through FoodNet Fast. This surveillance program allows users to select the pathogen they want to examine, as well as other characteristics, including test method, subgroup, year, age group, sex, race, and geographical location of cases reported to the CDC. These One Health surveillance programs provide data that helps to identify trends and patterns within foodborne pathogens.

It is well established that outbreaks of non-typhoidal human salmonellosis have been linked to consumption of contaminated animal products. However, human infections unrelated to food animal transmission, such as contaminated produce and other food types, environmental exposure, nosocomial, and community transmission, vectors, including house flies and cockroaches, as well as contaminated water sources, have been well documented (6, 15–17). It is also possible that unknown routes of *Salmonella* transmission to humans exist. This emphasizes the importance of ongoing surveillance, monitoring, and research of *Salmonella* transmission to humans.

In this study, we hypothesized based on secondary data that if food animals are the primary route of transmission of *Salmonella* species to humans, then there should be a correlation/similarity in the distribution and diversity of *Salmonella* serovars between food animals and humans. Our objectives are to explore the relatedness/similarity through the frequency distribution and diversity of *Salmonella* serovars from beef cattle before, at, and after slaughter in relation to human-derived serovars using statistical and epidemiological methods. This study may provide evidence that supports the availability of different and additional sources of human infections beyond food-animal sources.

## 2 Methodology and experimental design

### 2.1 Data source

The secondary data for our project was sourced from the historical and data repository collected by NARMS over the years in the United States. This data is readily available for public use and for secondary data analysis.^1^ This data underwent whole genome sequencing (WGS) and antimicrobial susceptibility testing (AST) by NARMS to identify each specific serovar and antibiotic susceptibility of *Salmonella* infection. *Salmonella* samples were isolated by NARMS between the years 1997 to 2023. More specifically, human samples ranged from 2002 through 2023, excluding years 2004, 2005, and 2006. Cecal samples ranged from 2013 through 2023, HACCP samples from 1997 through 2023, and retail meat samples from 2002 through 2021. The 3 cattle sources, cecal, HACCP, and retail, all excluded the year 2020. Unlike other study designs, our study considered data from a large time frame (1997–2023), which also represents more recent cases of *Salmonella* infection or contamination.

### 2.2 Epidemiological design

We used an ecological epidemiological study using the NARMS repository of data on *Salmonella enterica* collected as part of human surveillance, cattle, and cattle products (at cecal, HACCP, and retail meat) surveillance in the United States.

### 2.3 Methodology

**Figure 1:**
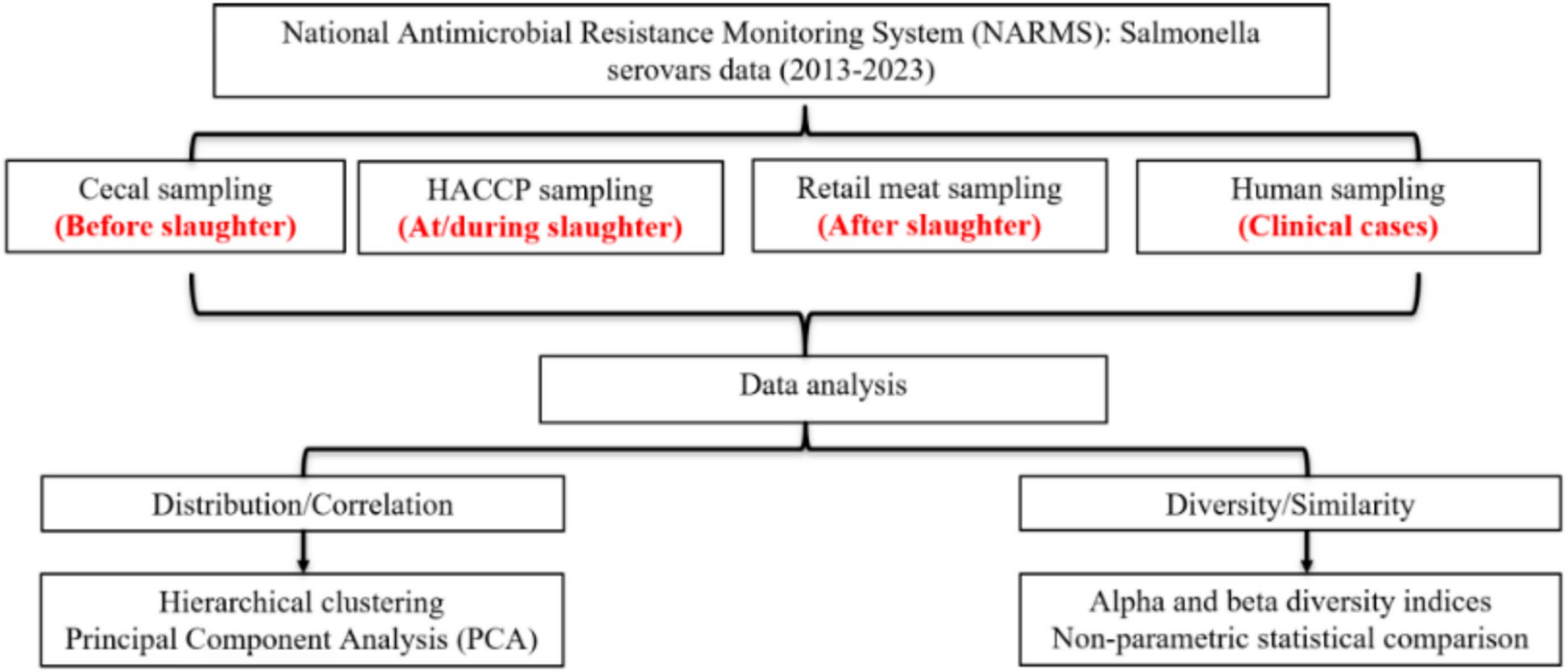
Methodology including data source and data analytical steps.

For this study, our methodology is represented by Figure 1. *Salmonella* surveillance data were obtained from the publicly available National Antimicrobial Resistance Monitoring System (NARMS) Integrated Data available through the U.S. Food and Drug Administration (FDA) NARMS Data Portal. The dataset was downloaded on 15 January 2025 and included all *Salmonella* isolates collected between 1997 and 2023 from the human, beef cattle, and retail meat surveillance programs. Only isolates with complete serovar information and a defined source were included in the analysis. Isolates with missing, incomplete, or unidentified serovar information were excluded. These isolated serovars were collected from human clinical cases (mostly from stool, blood, and urine) and at 3 different sampling phases of the beef animal processing: cecal (representing before slaughter), HACCP (at/during slaughter), and retail meat (after slaughter). Our focus was to explore the distribution and diversity of *Salmonella* serovars identified from human clinical cases and the 3 different phases of beef animal processing. We hypothesized that the distribution and diversity of *Salmonella* serovars from human clinical cases and beef animals would be similar. To test our hypothesis, hierarchical clustering method, principal component analysis, and alpha and beta diversity analyses were adopted as our statistical analytical framework. We performed hierarchical clustering and principal component analysis, factoring the period of the study (1997–2023) into our analyses. The period was categorized into 5 groups, including “1997–2001,” “2002–2006,” “2007–2011,” “2012–2016,” and “2017–2023.” These period categories were used as metadata for visualization and interpretation of the principal component analysis and hierarchical clustering, allowing assessment of temporal shifts in bacterial composition while improving sample size within each time interval and reducing random annual fluctuations. We conducted an agglomerative hierarchical clustering analysis to investigate the distribution of *Salmonella* serovars contingent on the prevalence and sampling sources over the 27-year surveillance period. The analytical matrix consisted of *Salmonella* serovars and their distributions across sampling sources. Agglomerative hierarchical clustering was performed to group *Salmonella* serovars according to the similarity of their distribution across sampling sources. The analytical matrix consisted of *Salmonella* serovars (rows) and their corresponding isolate counts across the sampling sources (columns). Before analysis, the data were centered and standardized using z-score transformation to ensure that all variables contributed equally to the clustering regardless of differences in scale. Euclidean distance was used to construct the dissimilarity matrix among serovars, and Ward’s minimum variance (Ward. D2) method was applied as the agglomerative linkage criterion to minimize within-cluster variance during cluster formation. The resulting hierarchical relationships among serovars were visualized using a dendrogram. Clusters were identified and interpreted based on the dendrogram structure and linkage distances, which represent the degree of dissimilarity among serovar profiles rather than their prevalence or frequency. Furthermore, to understand the prevalence and serovar distribution over the sampling sources, the most prevalent and widely distributed serovar clusters, based on the hierarchical clustering analysis, were subjected to principal component analysis (PCA). The relationship between the serovars and sampling source-period combination was further presented using PCA biplot and PCA projection as a heatmap. Principal component analysis is a method of reducing the dimensionality of a dataset and minimizing information loss to increase interpretability (18). Therefore, the variable and individual contributions of serovars and sampling sources to the principal component constructions will further provide more insight into the association and how *Salmonella* serovars are distributed with human cases and the 3 sampling sources of cattle over the surveillance period, as well as information on the most prevalent serovars. To explore the (dis)similarity in the diversity of *Salmonella* serovars over the sampling sources, we used the alpha and beta diversity indices for our analysis. Based on our hypothesis that if food animals are the primary route of *Salmonella* transmission to humans, then there should be a similarity in the diversity of *Salmonella* serovars isolated from beef cattle and human clinical cases collected by NARMS over the surveillance period. Therefore, we applied alpha and beta diversity indices, commonly used in ecological studies, to test our hypothesis. Firstly, we performed alpha diversity indices using Chao’s index, as a measure of serovar richness, and Shannon’s index, as a measure of both serovar richness and evenness. We statistically compare median alpha diversity indices between the sampling sources using Kruskal-Wallis’ test, adjusting for multiple comparisons using Bonferroni correction when necessary. Next, the Beta-diversity analysis of *Salmonella* serovars between the sampling sources was evaluated using the Bray–Curtis dissimilarity index. We visualized the beta diversity using a non-metric multidimensional scaling (NMDS) ordination plot. Analysis of Similarities (ANOSIM) and Permutational Analysis of Multivariate Analysis of Variance (PERMANOVA) were used to statistically explore beta diversity between sampling sources following a Permutational Analysis of Multivariate Dispersions (PERMDISP). Furthermore, we performed Principal Coordinates Analysis (PCoA) and Distance to centroid analysis as measures of multivariate dispersion and variability. We used similarity percentage (SIMPER) analysis to investigate the differential abundance of specific *Salmonella* serovars driving *Salmonella* community differences between the sampling sources (19). Specific serovars that accounted for the *Salmonella* community difference at a threshold percentage of 1% were investigated further using the Kruskal-Wallis (KW) non-parametric test adjusted for multiple comparisons using the Benjamin-Hochberg false discovery rate. For this study, data management was done using a Microsoft spreadsheet, and data analysis was conducted in the R computing environment (R version 4.4.1) and R packages including tidyverse package for data tidying, cluster package for agglomerative hierarchical clustering, while principal component analysis was performed using the FactoMineR and factoextra packages. For all our analyses, statistical significance was considered at *p* ≤ 0.05.

## 3 Results

### 3.1 Hierarchical clustering and PCA, and the period of study

**Figure 2:**
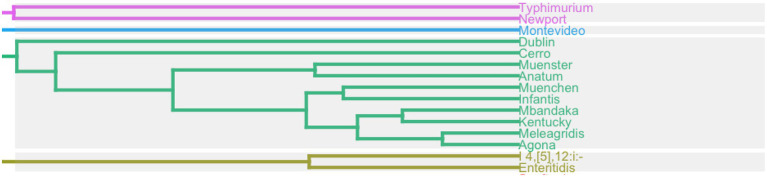
Cluster dendrogram for the first 4 clusters of *Salmonella* serovars from 4 sampling sources (cecal, HACCP, and retail cattle sources, and human clinical cases) over 27 years (1997–2023).

From the agglomerative hierarchical clustering, *Salmonella* serovars were grouped into 5 clusters based on their prevalence and distribution across sampling sources of the serovars and the period of the study. As a result of agglomerative hierarchical clustering, we identified the 15 most prevalent isolated *Salmonella* serovars from human, cecal, HACCP, and retail samples over the study period (Figure 2). These 15 serovars made up the first 4 clusters, while other serovars made up the 5th cluster (over 600 serovars, Supplementary Figure S1). Among the clusters, Montevideo serovar., in cluster 2, exhibited the largest hierarchical linkage distance among the serovars in the dendrogram, indicating that its temporal occurrence pattern differed substantially from the other serovars.

**Figure 3:**
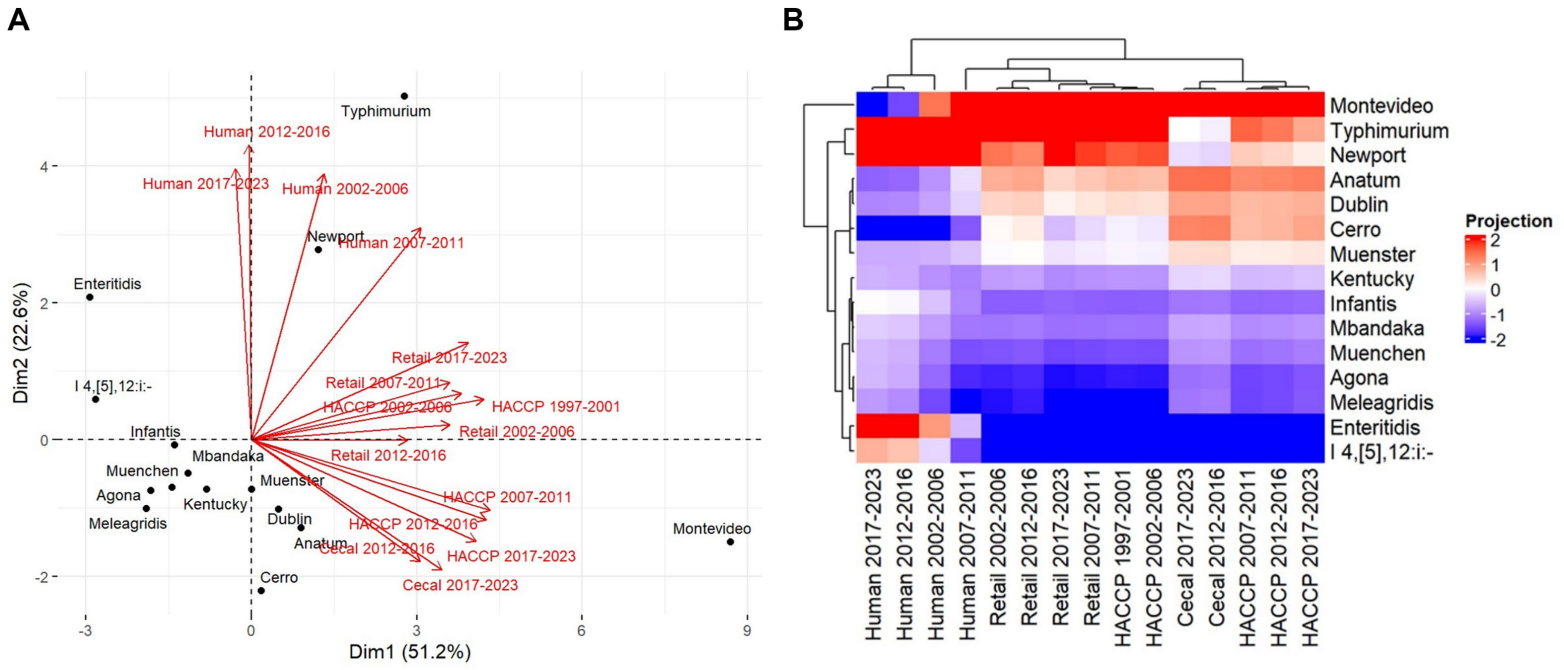
Principal component analysis (PCA) of *Salmonella* serovars from sampling sources and period of the study (1997–2023). **(A)** Showed the PCA biplot of *Salmonella* serovars and sampling sources-period. **(B)** Showed the heatmap of PCA projections of serovars onto the sampling source-period combinations.

From the PCA of the 4 clusters, PCA dimensions 1, 2, and 3 explained 82.6% of the data variance (Supplementary Figure S2). HACCP source and the 2007–2011 period are the top contributors for dimension 1 and for all 3 PCA dimensions combined. Human source and the 2012–2016 period contributed the most in dimension 2, and retail source and the 2012–2016 period contributed the most in dimension 3 (Supplementary Table S1; Supplementary Figure S2). Based on the sampling source/period and serovars’ contributions to the PCA dimension constructions, *Salmonella* serovar Montevideo contributed the most to dimension 1 and all three dimensions combined (Supplementary Figure S3). Typhimurium contributed the most to dimension 2, and Dublin contributed the most to dimension 3. From the PCA biplots and projection (Figure 3), Typhimurium and Newport serovars aligned strongly with human clinical, retail, and HACCP sources. Serovars Enteritidis and monophasic Typhimurium (I 4,[5],12:i:-) were associated with human clinical cases in the more recent period (2012–2023) compared to earlier years (2002–2011), when Montevideo predominated. Serovar Montevideo was disseminated and associated with Cecal, HACCP, and retail sources irrespective of the sampling period. Serovars Dublin, Cerro, and Anatum were primarily associated with cattle sources irrespective of the period of the study.

### 3.2 *Salmonella* serovar diversity across the sampling sources and period of the study

**Figure 4:**
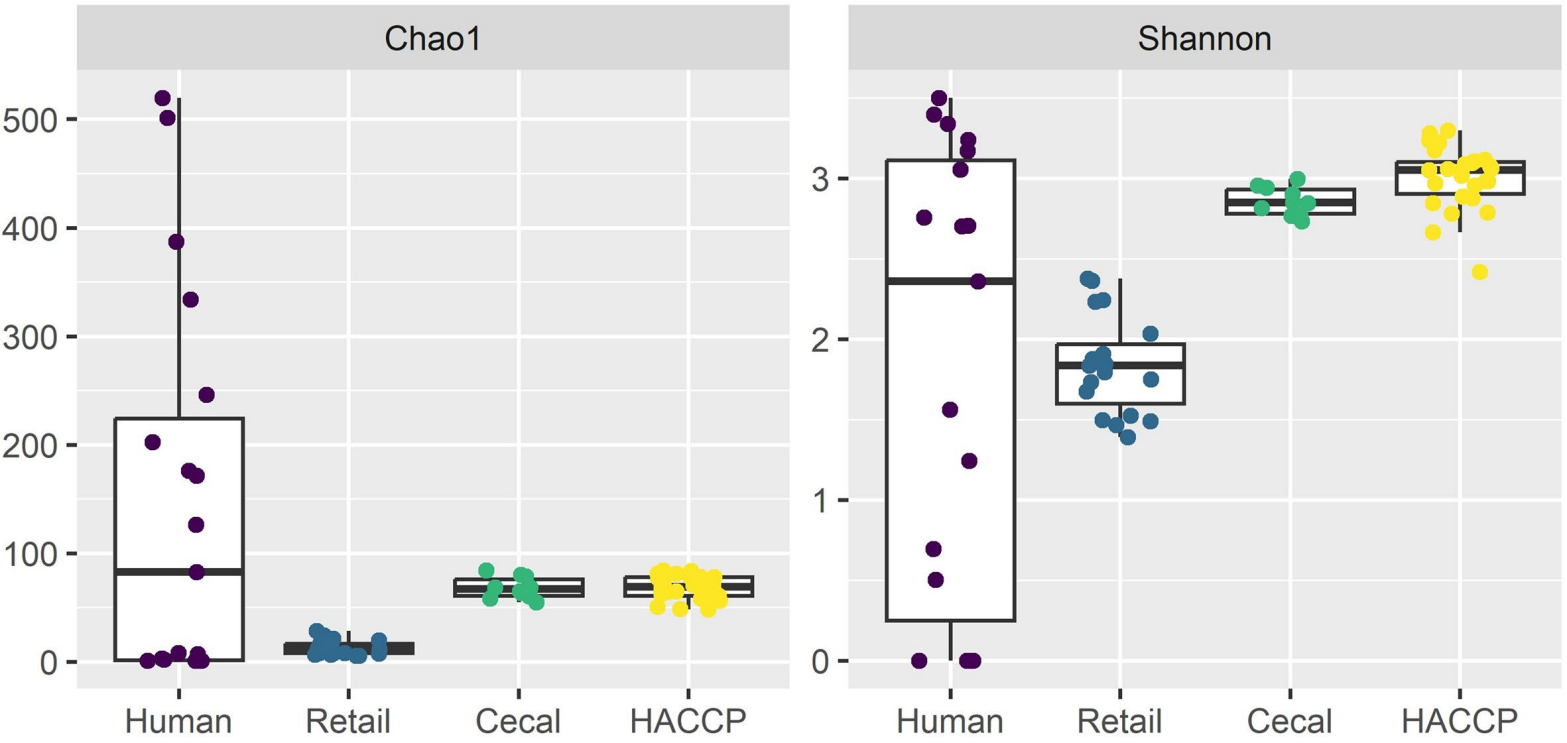
Chao1 richness and Shannon diversity index of *Salmonella* serovars by sampling sources (1997–2023).

Using the Chao1 serovar richness (Figure 4), the median serovar richness was statistically significantly different between sampling sources (*p* < 0.001). Post-hoc analysis, adjusting for multiple comparisons, showed no statistically significant difference in median (IQR) serovar richness between the human sources (82.5(222.6)) and other cattle sampling sources, including Cecal (66.7(15.2), *p* = 1.00), HACCP (69(17.4), *p* = 1.00), and retail sources (11.0(8.6), *p* = 1.00). Conversely, median (IQR) serovar richness is statistically significantly higher in cecal sources compared to the retail sources (*p* < 0.001). Similarly, median serovar richness is statistically significantly higher in HACCP sources compared to the retail sources (*p* < 0.001). Using the Shannon diversity index as a measure of both richness and evenness of *Salmonella* serovars across the sampling sources and period of the study (Figure 4). For this study, there was a statistically significant difference in the median Shannon index between the sampling sources (*p* < 0.001). Following post-hoc analysis adjusting for multiple comparisons, the median (IQR) Shannon diversity index was statistically significantly higher in HACCP sources (3.1(0.2)) compared to each of cecal (2.9(0.2), *p* = 0.011), retail sources (1.8(0.37), *p* < 0.001), and human sources (2.4(2.9), *p* = 0.026). Also, the median Shannon diversity index was statistically significantly higher in cecal sources compared to the retail sampling sources (*p* < 0.001).

**Figure 5:**
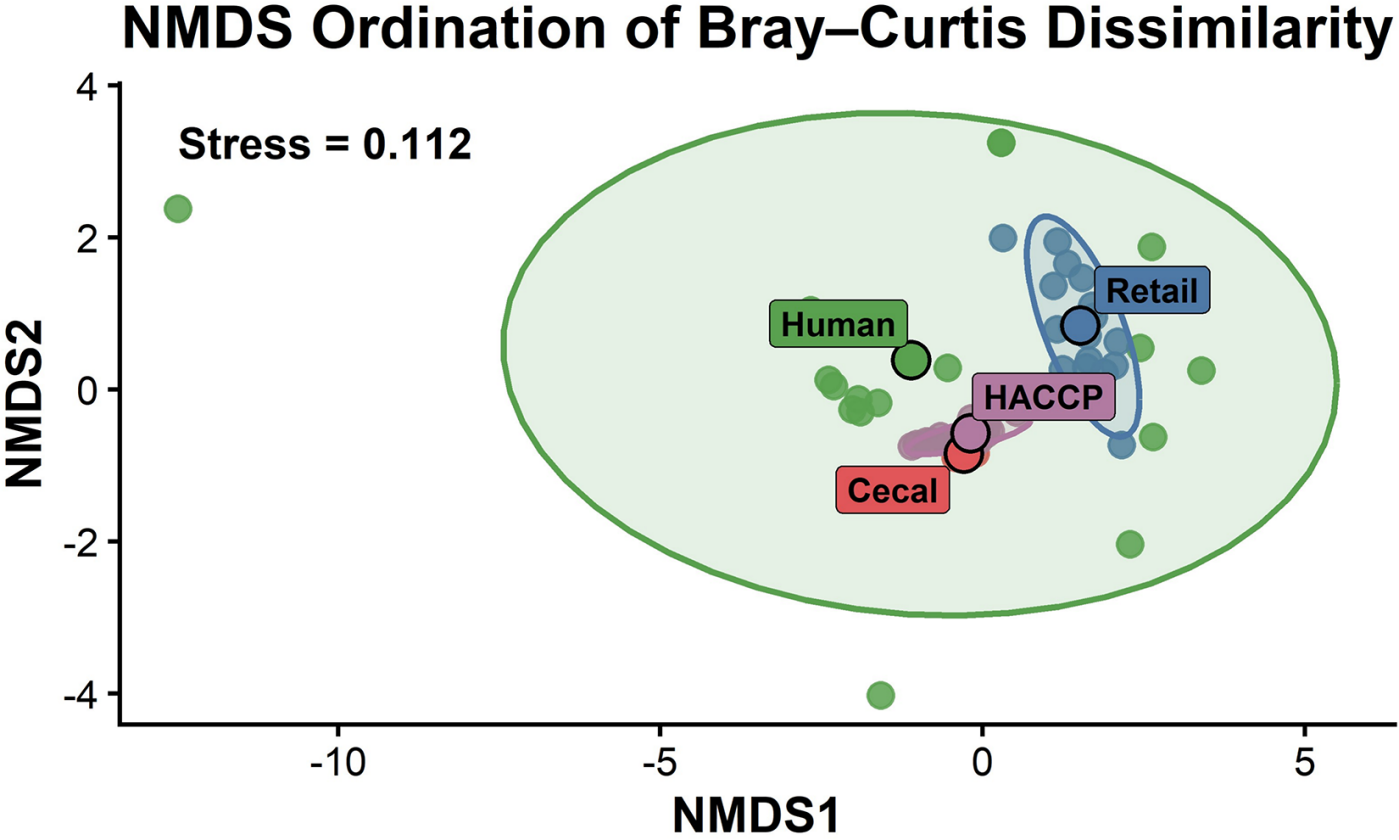
Non-multidimensional scaling (NMDS) plot of the Bray–Curtis dissimilarity for *Salmonella* serovar community differences between the sampling sources. Each dot represents the serovar community of each period of the study. Ellipses represent the standard error around the centroid of each sampling source and are colored by sampling source. *Salmonella* serovar community composition between the sampling sources differs and is distinct (ANOSIM, *p* < 0.001). Stress = 0.112 indicates an excellent to good fit and shows that the NMDS plot reliably captures the underlying structure and relationships between the *Salmonella* serovar community and sources without significant distortion.

For the beta diversity, the NMDS plot (Figure 5) revealed distinct clustering of *Salmonella* serovars by sampling sources. Retail serovars formed a tight cluster, partially overlapping with human clinical sources, consistent with the role of retail meat as a major pathway for human salmonellosis. Human sources, however, were more dispersed, reflecting their greater serovar diversity and potential contributions from additional sources. HACCP and cecal serovars clustered closely together, suggesting that regulatory monitoring programs capture serovars representative of pre-harvest cattle reservoirs. Overall, the NMDS plot reinforces the ecological segregation of *Salmonella* serovars by source, while highlighting the likelihood of transmission between retail meat and human clinical sources. PERMDISP analysis revealed significant differences in within-source variability of *Salmonella* serovar compositions. Cecal and HACCP isolates were the most homogeneous, retail isolates showed intermediate variability, and human isolates were the most heterogeneous. The Principal Coordinates Analysis (PCoA) of multivariate dispersion showed that Cecal and HACCP isolates formed tight clusters around their group centroids, indicating consistent serovar structures (Supplementary Figure S4). Retail isolates formed a distinct but moderately dispersed cluster, whereas human isolates exhibited the greatest dispersion, confirming their broader serovar diversity. Distance-to-centroid analyses (Supplementary Figure S5) reinforced these patterns, with significantly lower dispersion in Cecal and HACCP isolates compared to Retail and Human isolates. These findings were further supported by the ANOSIM analysis. Analysis of similarities (ANOSIM) demonstrated significant differences in *Salmonella* serovar compositions among the sampling sources (R = 0.512, *p* < 0.001), with between-source differences exceeding within-source variation (Supplementary Figure S6). The ANOSIM analysis further supports the NMDS plot, indicating the *Salmonella* serovar community was distinct between the sampling sources. Cecal and HACCP serovars were homogeneous in their serovar profiles, while retail serovars exhibited intermediate variability, and human serovars were the most diverse and heterogeneous community. R-square from PERMANOVA showed that the sampling sources explained 39% of the variation in the *Salmonella* serovar community. Together, ANOSIM and PERMDISP results indicate that *Salmonella* serovar distributions are strongly structured by the sampling source and distinct serovar community compositions.

**Figure 6:**
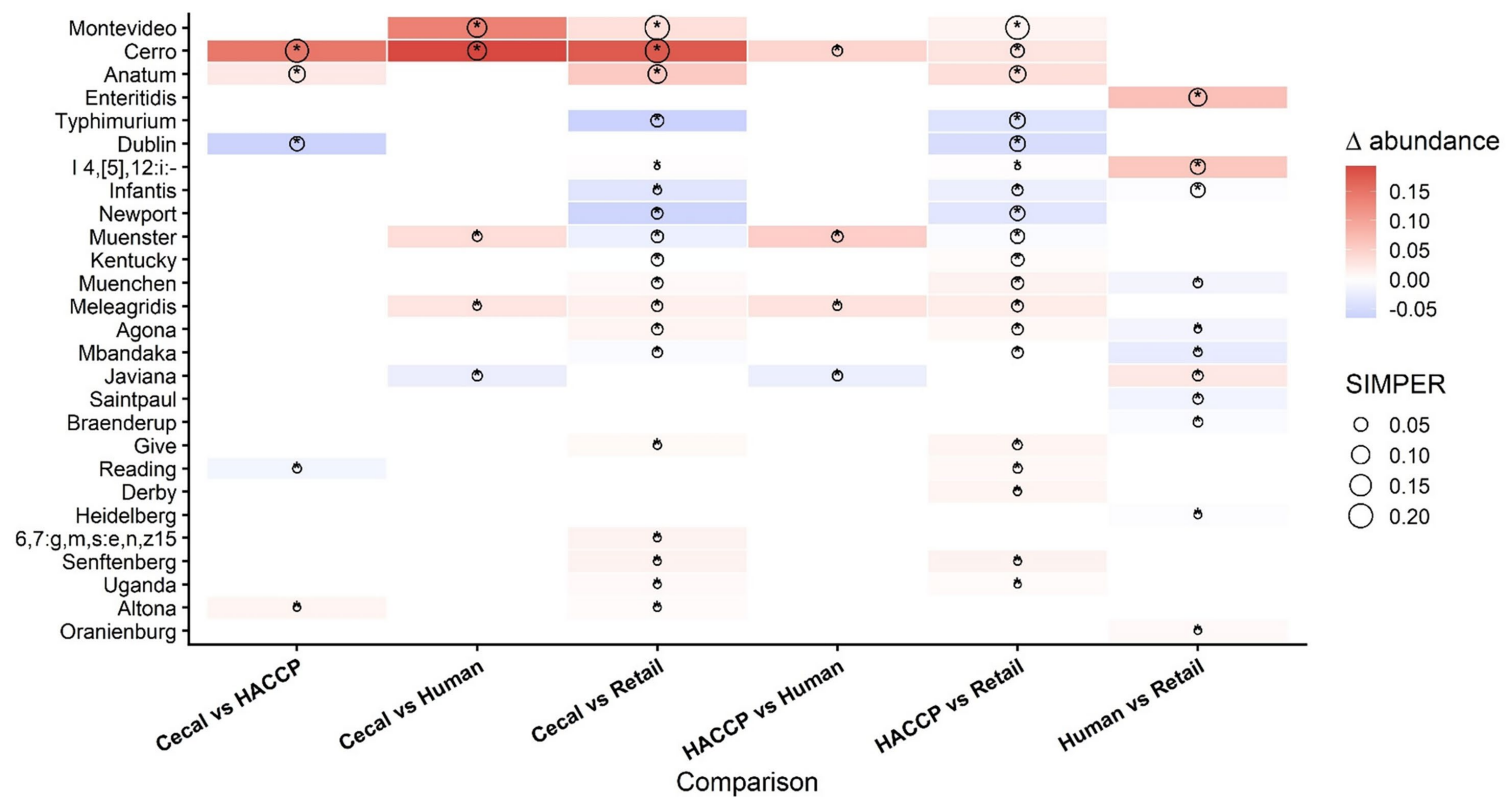
Differential abundance of *Salmonella* serovars between sampling sources using similarity percentage analysis. Only statistically significant differences in the abundance of *Salmonella* serovars between the sampling sources were shown. The label indicates *Δ* = mean(Left) − mean(Right) in the serovars abundance between the sources, and * indicates FDR < 0.05. Tile color shows the direction and magnitude of abundance difference; circle size shows SIMPER contribution.

Using the Similarity percentage analysis, we identified serovars contributing differentially to the dissimilarities among the sampling sources (Figure 6; Supplementary Table S3). Comparisons between cecal and retail isolates showed that serovars Cerro, Montevideo, and Anatum were enriched in cecal samples, whereas Typhimurium and Newport were significantly more abundant in retail sampling sources. Similarly, compared to retail sampling sources, serovars Cerro, Montevideo, and Anatum were enriched in HACCP sources, while serovars Typhimurium, Dublin, Infantis, and Newport were enriched in retail sources. However, compared to the HACCP source, serovars Cerro and Anatum were enriched in cecal sources, while serovars Dublin, Reading, and Altona were enriched in HACCP sources. Consistently, serovars Montevideo, Cerro, Meleagridis, and Muenster were enriched as cecal serovars compared to the Javiana serovar in human sources. Also, in comparison to human sources, serovars Cerro, Muenster, and Meleagridis were differentially enriched in HACCP sources, while serovar Javiana was enriched in the human sources. Human sources were differentiated from retail sources by higher relative abundances of Enteritidis, Javiana, and the monophasic variant I 4,[5],12:i:-, while retail sources were more enriched for Infantis and Saintpaul (Figure 6).

## 4 Discussion

This study provides evidence demonstrating that *Salmonella* serovar distributions follow distinct ecological patterns structured by sampling source, with these patterns remaining consistent across multiple multivariate analytical approaches adopted in this study. Ordination analyses (PCA and MDS) revealed distinct clustering of isolates by sampling source, with significant differences in both serovar associations and within-source diversity. These findings illustrate the transmission landscape of *Salmonella* serovars, where different sampling sources are represented by distinct *Salmonella* serovar niches with varying degrees of diversity.

Using PCA, the most common *Salmonella* serovar isolated within our sampling groups (Cecal, HACCP, Retail, and Human) was *Salmonella* Montevideo. This finding is in line with another study, which found that *Salmonella* Montevideo was identified to be one of the most common serovars detected among beef sources (20). This discovery can be due to the survivability of *Salmonella* within cattle, contaminated environments, and cattle sources that spill over to humans. *Salmonella Typhimurium* followed in prevalence after Montevideo. As cases of *Salmonella* Newport are rising in humans and animals, data suggest that a possible source of infection includes dairy cattle through routes of exposure such as unpasteurized milk, sick cattle, and dairy farm environments (21). According to a study in France, *Salmonella* Montevideo was the most prevalent serovar recorded among cattle, which they noted was also well documented among dairy animals (22). In a different study, *Salmonella* Newport was detected, via feces, as the most prevalent serovar among dairy cattle in the United States and was also highly multidrug resistant (23). As previously stated, these *Salmonella* serovars can be spread to humans from cattle through direct and indirect exposures such as contaminated environments, contaminated food products, and sick animals (22).

Principal component analysis identified serovar Montevideo as the largest contributor to Dimension 1, accounting for 65.52% of the variation explained by that principal component, whereas serovar Typhimurium contributed most to Dimension 2 (49.63%). These findings indicate that the distributions of Montevideo and Typhimurium were major factors influencing the separation of samples along the first and second principal components, respectively. Because principal components are mathematical constructs that summarize variation in multivariate data, these results should be interpreted as reflecting patterns of serovar distribution rather than underlying biological mechanisms. Nevertheless, the prominent contribution of Montevideo to Dimension 1 is noteworthy, as previous studies have reported that this serovar is frequently recovered from beef processing environments and cattle production systems in the United States (24). Likewise, the strong contribution of Typhimurium to Dimension 2 is consistent with published surveillance studies demonstrating its frequent occurrence in humans and its association with cattle (25). Dublin contributed the most to dimension 3 (52.44%), which corroborates another study that identified Dublin as a bovine-adapted pathogen that is capable of transmitting from cattle to humans (26). These serovars all contribute to their respective dimensions according to various characteristics, including transmission pathway (from source to source) and species specificity. As stated previously, the total cumulative variance for dimensions 1–3 together is 82.6%. Our PCA biplots gave a detailed and visualized example of the correlations between *Salmonella* serovars and sources. In dimensions 1 to 2, Typhimurium, Newport, Enteritidis, and I 4,[5], 12:i:- were driven by humans. These serovars, also referred to as DENT (*Salmonella* serovars Dublin, Enteritidis, Newport, Typhimurium, and I 4,[5], 12:i:-), are recognized by the USDA Agricultural Research Service and others as highly pathogenic *Salmonella* (HPS) (27). These same serovars, along with others, are also recognized as invasive nontyphoidal *Salmonella* (iNTS), which are more virulent, causing more severe illness and a higher chance of hospitalization (28). These HPS serovars have also been identified as one of the most prevalent pathogens in humans (29). The other sources were driven by Montevideo, which we discussed as having a strong correlation with beef processing units and cattle environments. For dimensions 2 to 3, comparable results were shown for humans. Humans once again were the primary drivers of *Salmonella* serovars Typhimurium, Newport, Enteritidis, and I 4,[5], 12:i:-. However, the other serovars were driven by different sampling sources. *Salmonella Cerro* was driven by the cecal source. Similarly, a study in the United States found *Salmonella* Cerro as one of the most prevalent serovars, shed through feces among cow-calf operations (30). However, they also identified *Salmonella* Oranienburg as most prevalent alongside Cerro, both representing 21.8% of positive isolates (30). Next, the Montevideo serovar was driven by the HACCP source, consistent with a previous report where Montevideo was the most prevalent among beef samples (22). Furthermore, *Salmonella* Dublin, which is a host-adapted serovar to cattle, was driven by retail sources consistently with findings from a previous report involving beef cattle meat (31).

With the sampling period factored into the analysis, serovars Typhimurium, Newport, and monophasic Typhimurium I 4,[5], 12:i:- have replaced Enteritidis as the leading cause of human salmonellosis in recent times. Meanwhile, Dublin and Infantis are linked to retail meat sources, most especially before the year 2017, while serovars associated with cecal sources were stable irrespective of the sampling period. These findings suggest the need for sampling source-specific surveillance and targeted interventions addressing the epidemiological shift in serovars of human health concern, while addressing persistent reservoirs of certain serovars at the farm level.

Using diversity indices, we observed that there was more variability within the human source than the other sampling sources. The ecological structure revealed by our analysis showed a clear hierarchy of diversity across the beef-to-human transmission pathways. Human clinical serovars were dominated by Typhimurium, Newport, and Enteritidis, consistent with their established role as leading causes of human salmonellosis (32, 33). However, human-associated serovars also displayed the greater variability in ordination, as confirmed by PERMDISP analyses, suggesting that multiple ecological and transmission pathways contribute to the serovar diversity observed in clinical cases (34). This high variability in human-associated serovars reflects the complex epidemiological reality that humans are exposed to *Salmonella* through various transmission routes, including food animal sources, nosocomial and community transmission, environmental exposures, global travel, and other unknown sources, as well as other food sources beyond food animals (35, 36). Notably, Montevideo and Infantis were strongly associated with retail and HACCP samples, indicating their importance in the peri- and post-harvest environment (37, 38).

In contrast to the diversity seen in humans, cattle-associated sources showed notable stability. Cecal and HACCP isolates were the most homogeneous, clustering closely together in the alpha diversity and PCoA analyses and showing significantly lower dispersion in PERMDISP. These cattle-associated sources highlight a narrower and more stable subset of serovars typical of cattle reservoirs, such as Cerro, Anatum, Kentucky, Mbandaka, Muenchen, Meleagridis, and Muenster, irrespective of the period of study (39, 40). This emphasizes the importance of monitoring programs such as HACCP in identifying important critical control points from farm to table and in achieving a better understanding of *Salmonella* ecology in pre- and peri-harvest food safety.

The statistical validation of this separation through ANOSIM analysis (R = 0.512, *p* < 0.001), further confirmed that sources differ and are distinct both in their serovar composition and in their internal heterogeneity (41). Specifically, Cecal and HACCP sources reflect consistent pre-harvest and peri-harvest serovar pools that capture a narrow and limited subset of *Salmonella* serovars community typical of cattle reservoirs. However, human sources represent the broadest ecological niche of *Salmonella* serovars, consistent with multiple exposure pathways beyond food animal sources, while retail sources occupy an intermediate position, bridging homogeneous pre-harvest reservoirs and heterogeneous human clinical sources. The consistent alignment of human and retail isolates highlights the significance of strengthening interventions along the farm-to-fork continuum in reducing the burden of meat-borne salmonellosis. However, the broader diversity of human-associated serovars may suggest exposure to rare *Salmonella* serovars and potential roles of other sources beyond food animal sources that warrant further and additional evidence (34, 37).

Lastly, using SIMPER analysis, we showed which *Salmonella* serovars contribute to and drive the differential abundance between the sampling sources, as well as provide an indication of likely and unlikely transmission direction potential. For instance, *Salmonella* Montevideo contributed the highest (20.8%) to cecal-retail source differences, while Monophasic Typhimurium contributed the least (1%) (Figure 6). Similarly, *Salmonella* Montevideo contributed the highest (18.8%) to HACCP-retail, while Monophasic Typhimurium contributed the least (1%) (Figure 6). The comparison of cecal-HACCP results in *Salmonella* Cerro contributing the highest (17.8%) and Altona contributing the least (1.3%) (Figure 6). The human-cecal comparison explains how the highest contributor is Montevideo (11.7%), while the lowest is Meleagridis (1.5%) (Figure 6). HACCP-human reflects a similar result of Meleagridis being the lowest (1.7%), but Muenster represents the highest (3%) (Figure 6). The last comparison, human-retail, showed Enteritidis as the highest contributor (9.4%), while Oranienburg was the lowest (1.2%) (Figure 6). The comparison between human and cecal *Salmonella* serovars shows that Montevideo and Cerro were statistically and significantly more abundant in cecal samples compared to human samples. Cerro is likely not a reservoir of human sources since the differential abundance of humans is near 0%. Therefore, this finding may suggest little to no likelihood of risk of this serovar to humans from cecal sources where Cerro is differentially more abundant. As for Montevideo, there may be little transmission to the human source, which has a differential abundance of 7.25%. Serovars Muenster and Meleagridis have cecal differential abundances of 3.79 and 2.77%, respectively (Supplementary Table S3). Both of those serovars have a human abundance of near 0%, indicating that the transmission of those *Salmonella* serovars from the cecal route to humans is unlikely. Similarly, in the Human-HACCP comparison, *Salmonella* serovars Muenster and Cerro are statistically and significantly more abundant in HACCP samples compared to human samples. The comparison between HACCP and humans showed minimal to no transmission of *Salmonella* serovars Muenster, Cerro, and Meleagridis to humans. Serovars Muenster, Cerro, and Meleagridis have HACCP differential abundances of 5.41, 4.59, and 3.06%, respectively, while the human source is near 0% for all 3 (Supplementary Table S3). This indicates that transmission of these serovars from HACCP to humans is less likely to occur. Across all cattle sources, there was an unlikely transmission of *Salmonella* Javiana to humans. Therefore, Javiana represents a key example of non-beef transmission pathways, showing differential abundances of 0% in both cecal and HACCP samples, indicating that humans acquire serovar Javiana from sources other than cattle. However, previous studies have shown that serovar Javiana can be transmitted to humans through contaminated fresh products, herbs, water, and contact with animals (26). Finally, the comparison between humans and retail meat indicates that *Salmonella* serovars Enteritidis and I 4,[5],12:i:- are statistically and significantly more abundant in human samples, and Infantis is statistically and significantly more abundant in retail samples. Their differential abundance values are 8.86, 6.86, and 5.37%, respectively (Supplementary Table S3). There are indications of transmission from retail Infantis to humans, shown by their high and similar differential abundances, as well as Braenderup, Heidelberg, Muenchen, and Saintpaul. There is little indication of transmission from retail Agona and Mbandaka to humans. Serovars Enteritidis, I 4,[5],12:i:-, Javiana, and Oranienburg are statistically and significantly more abundant for humans than retail, which indicates no transmission from retail to humans, but potential transmission from other sources.

There were some limitations in our study. Sample sizes for each sampling source were very uneven, and this might have introduced sampling bias. With the human sample size totaling 152,659 and the retail meat sample size totaling 254, there is a limitation due to the over-representation of human *Salmonella* serovars, which can influence the serovar diversity and distribution. All the samples were collected from 1997 to 2023; however, not every source had samples from each of those years. Each of the 4 sources consists of samples within a different range of years. This is a limitation because these collections may have been taking place during different outbreaks. The year 2020 was completely excluded from all 4 sample sources, with the assumption that NARMS could not take samples during the COVID-19 Pandemic. Another limitation includes many serovars being labeled as “unknown,” “undetermined,” etc., which were removed from our data due to uncertainty. Also, genomic analyses were not considered in this study, which also limits this study in establishing any transmission direction or dynamics between cattle and human sources. Genomic sequencing would be valuable for confirming epidemiological relationships suggested by serovar distributions.

Future studies will replicate this work in other food-producing animals, like poultry and swine, to broaden our understanding of *Salmonella* epidemiology and ecology within the one health framework. This research may also enhance the overall understanding of *Salmonella* transmission pathways and assist in identifying the major sources of *Salmonella* to humans. By understanding these transmission patterns, effective measures can be implemented to prevent and control *Salmonella* transmission to humans and reduce its occurrence in food animal production. Also, future studies are needed to continue providing scientific data about the exposure and transmission of *Salmonella* to humans from other sources unrelated to food animal sources.

## 5 Conclusion

As *Salmonella* remains a great concern to public health, it is important to have a clear understanding of transmission patterns to control and prevent future *Salmonella* infection and contamination. This study aimed to explore the relatedness and similarity through the diversity and frequency distribution of *Salmonella* serovars from beef cattle before, at, and after slaughter in relation to human-derived serovars. We concluded that in addition to food animal sources, there may be sources other than beef cattle contributing to the transmission of *Salmonella* to humans. According to our results from beta diversity, we found that *Salmonella* serovar communities between beef cattle and human sources were ecologically distinct, with stabled cattle-associated serovars, while human serovars showed greater diversity. Indicating there are additional pathways of transmission to humans unrelated to beef cattle.

## Data Availability

The original contributions presented in the study are included in the article/Supplementary material, further inquiries can be directed to the corresponding author.

## Footnotes

1
https://www.cdc.gov/narms/index.html
