# Supplementary material for: Distribution and diversity of Salmonella serovars from beef cattle before, at, and after processing in relation to human-derived serovars
Source: Front Vet Sci. 2026 Jul 22;13:1897484. doi: 10.3389/fvets.2026.1897484 (PMC13437263; doi:10.3389/fvets.2026.1897484)
Supplement: Supplementary file 1 [file Data_Sheet_1.PDF]

Supplemental Material

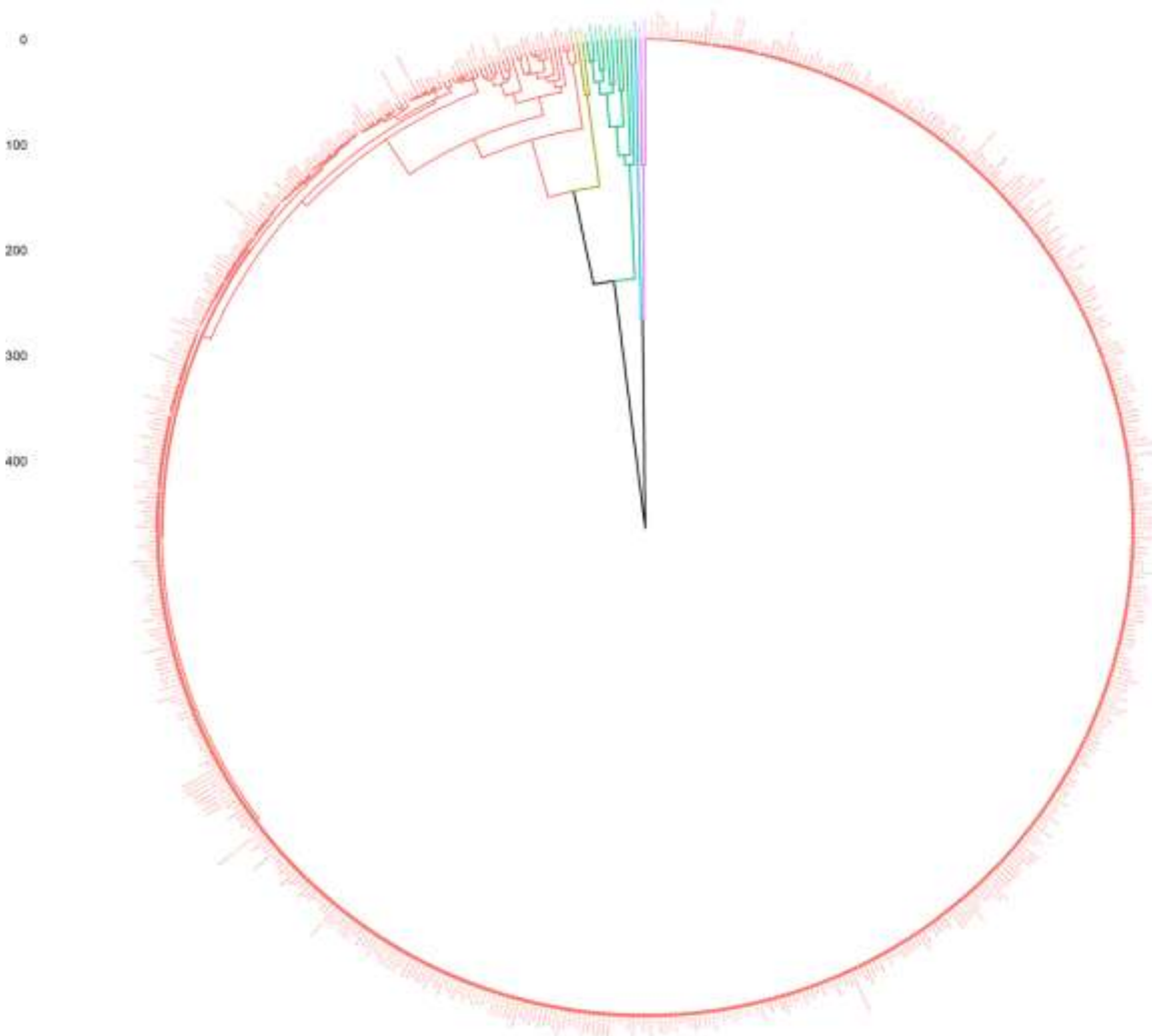

Figure S1: Agglomerative hierarchical clustering for Salmonella serovars, including the period of the study as a factor. The first 4 clusters represent the top 15 most prevalent *Salmonella* serovars

Table S1. Sampling sources and period contributions to principal components for different sampling sources and periods of study.

	Dim.1	Dim.2	Dim.3	Dim.4	Dim.5
Cecal 2012-2016	5.59	4.32	26.01	0.55	0.01
Cecal 2017-2023	7.13	4.93	17.71	1.35	1.43
HACCP 1997-2001	10.58	0.47	3.08	7.75	0.06
HACCP 2002-2006	8.66	0.62	0.11	31.50*	0.08
HACCP 2007-2011	11.17*	1.45	3.73	1.81	0.18
HACCP 2012-2016	10.84	1.87	0.69	8.72	0.03
HACCP 2017-2023	9.90	2.99	0.15	5.62	1.33
Human 2002-2006	1.04	20.40	0.32	8.22	5.91
Human 2007-2011	5.62	12.94	1.70	0.16	11.80
Human 2012-2016	0.00	25.11*	1.48	9.54	1.07
Human 2017-2023	0.05	21.21	0.18	10.29	23.14*
Retail 2002-2006	7.70	0.06	10.35	4.41	21.00
Retail 2007-2011	7.74	0.95	6.05	1.23	8.18
Retail 2012-2016	4.77	0.00	26.73*	5.29	21.59
Retail 2017-2023	9.20	2.69	1.70	3.56	4.19

*The most important variable for contribution to each dimension

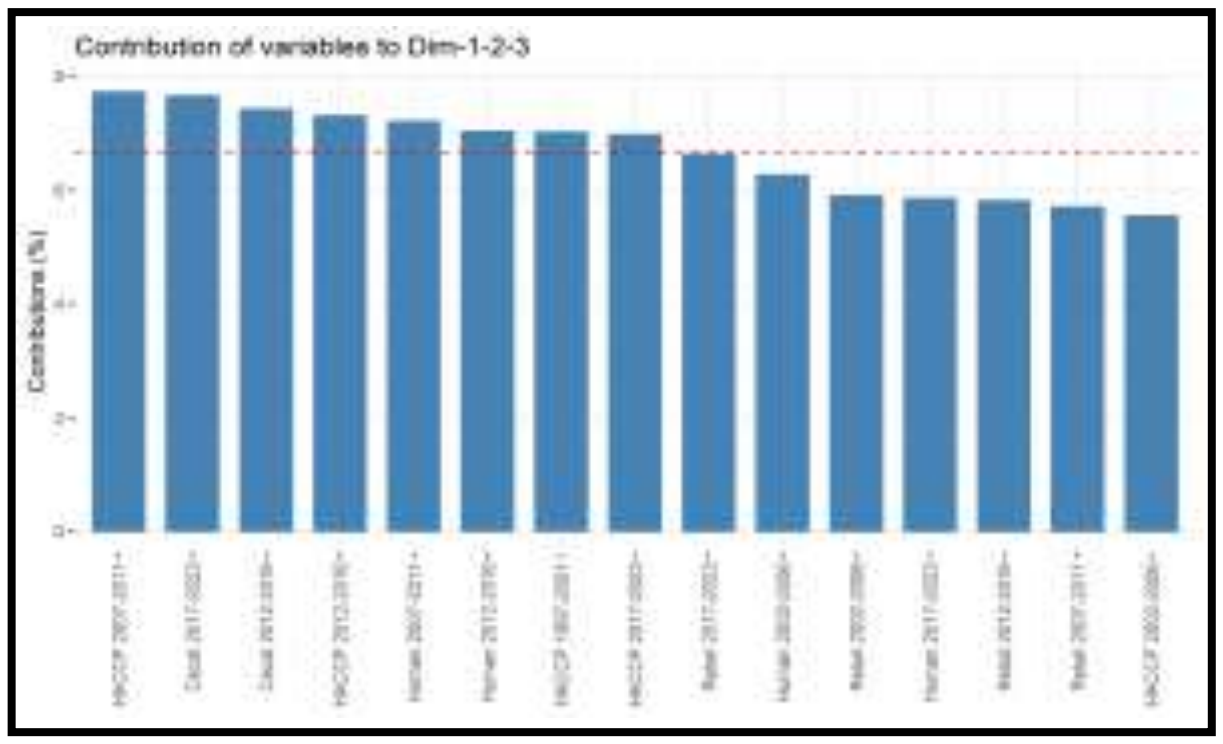

Figure S2a Contribution of variables to Dim-1-2-3

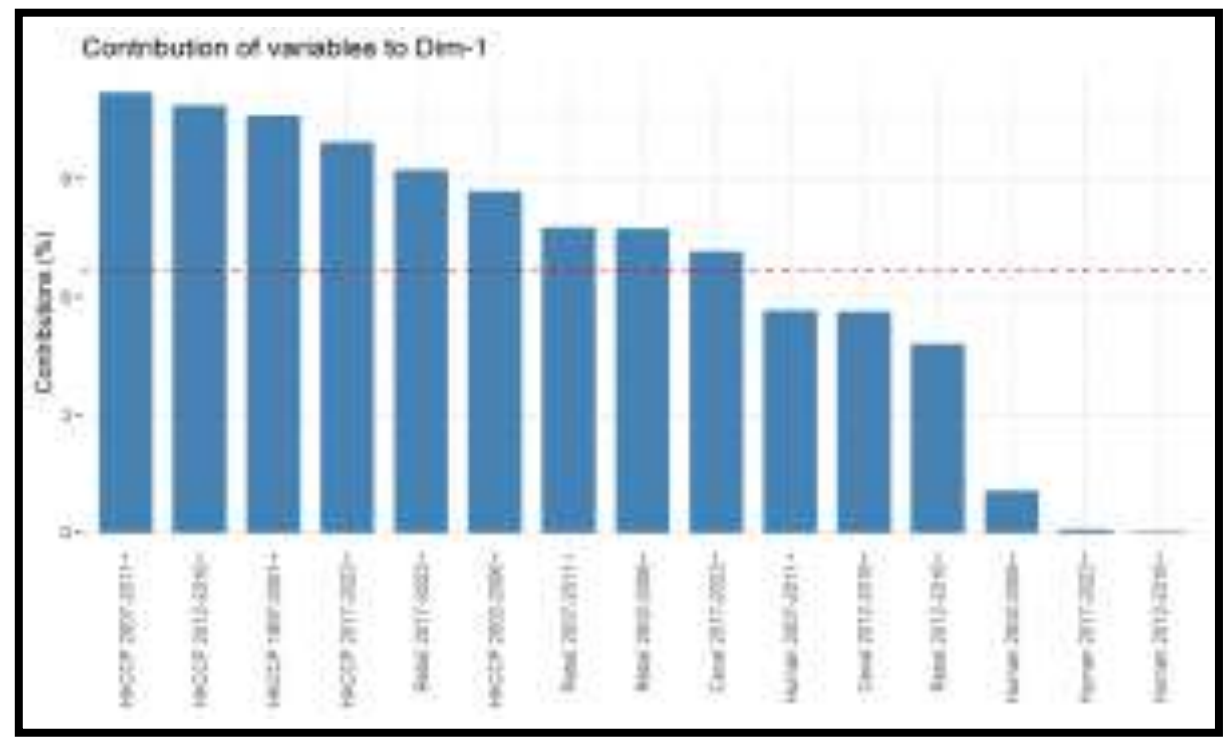

Figure S2b Contribution of varibales to Dim-1

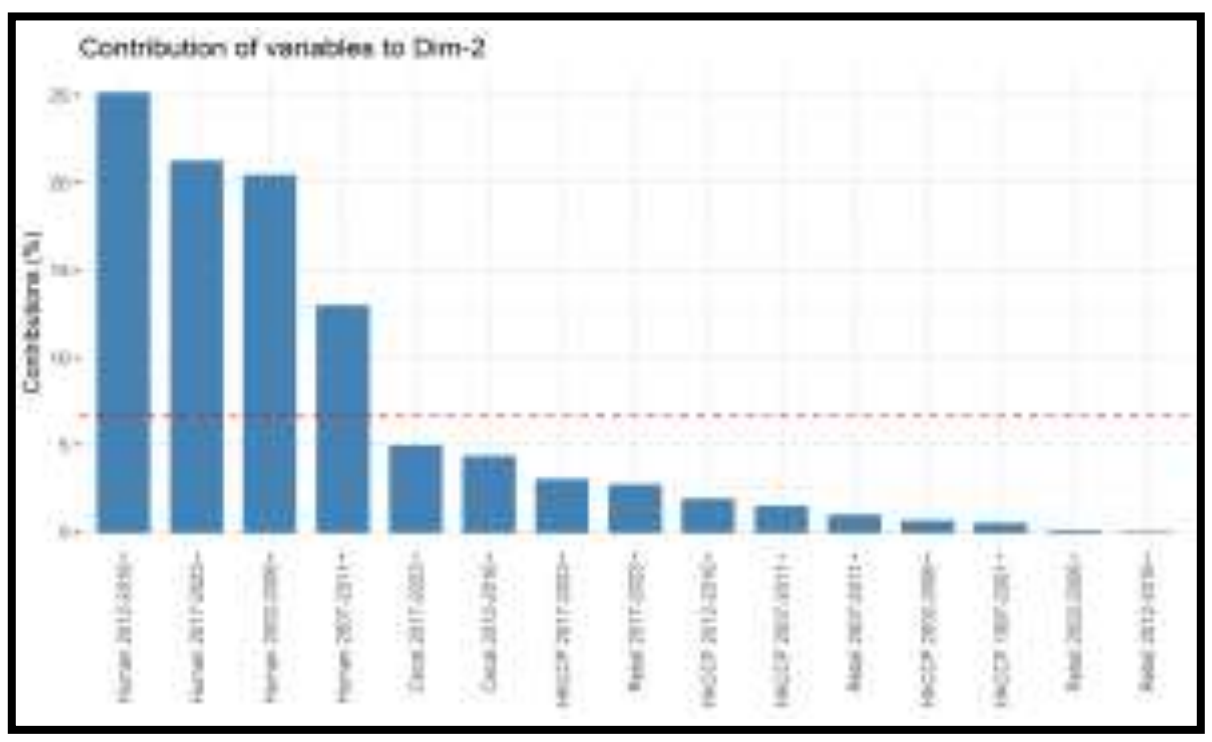

Figure S2c Contribution of variables to Dim-2

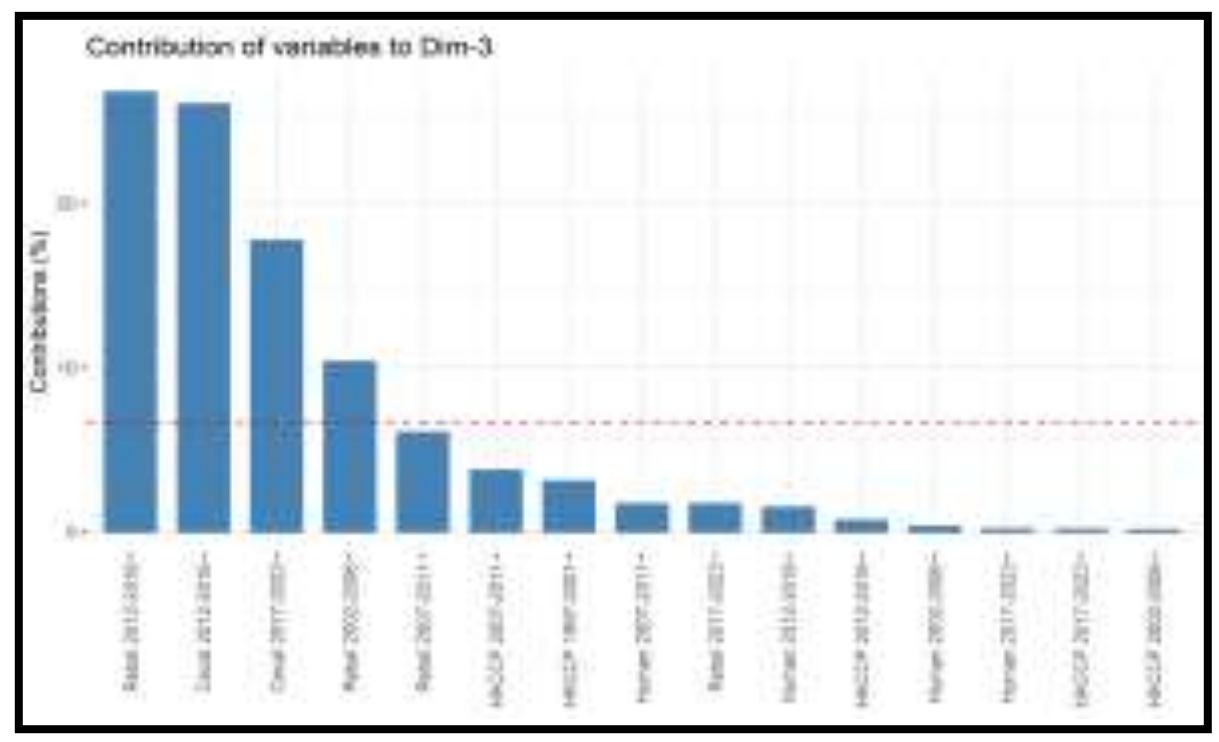

Figure S2d Contribution of variables to Dim-3

Figure S2: Sampling sources and period contributions to principal components for different sampling sources and periods of study. Individuals measuring above the red line indicate those who exceed the average contribution expectation.

Table S2: Salmonella serovars' contributions to principal components for different sampling sources and periods of study.

	Dim.1	Dim.2	Dim.3	Dim.4	Dim.5
Agona	2.89	1.08	0.08	3.13	3.61
Anatum	0.70	3.29	3.08	7.30	0.17
Cerro	0.03	9.58	33.64	2.30	1.92
Dublin	0.21	2.03	52.44*	5.18	9.60
Enteritidis	7.44	8.55	0.07	16.94	33.38*
I 4[5],12:i:-	6.90	0.69	0.21	7.09	1.13
Infantis	1.68	0.01	2.07	2.64	1.06
Kentucky	0.57	1.03	0.08	0.99	2.76
Mbandaka	1.14	0.48	1.19	6.75	1.14
Meleagridis	3.13	2.01	0.26	0.13	3.70
Montevideo	65.52*	4.41	0.02	6.73	6.25
Muenchen	1.81	0.95	0.74	4.35	0.37
Muenster	0.00	1.03	1.31	24.80*	10.29
Newport	1.28	15.22	1.12	11.66	4.35
Typhimurium	6.69	49.63*	3.69	0.00	20.28

*The most important variable for contribution to each dimension

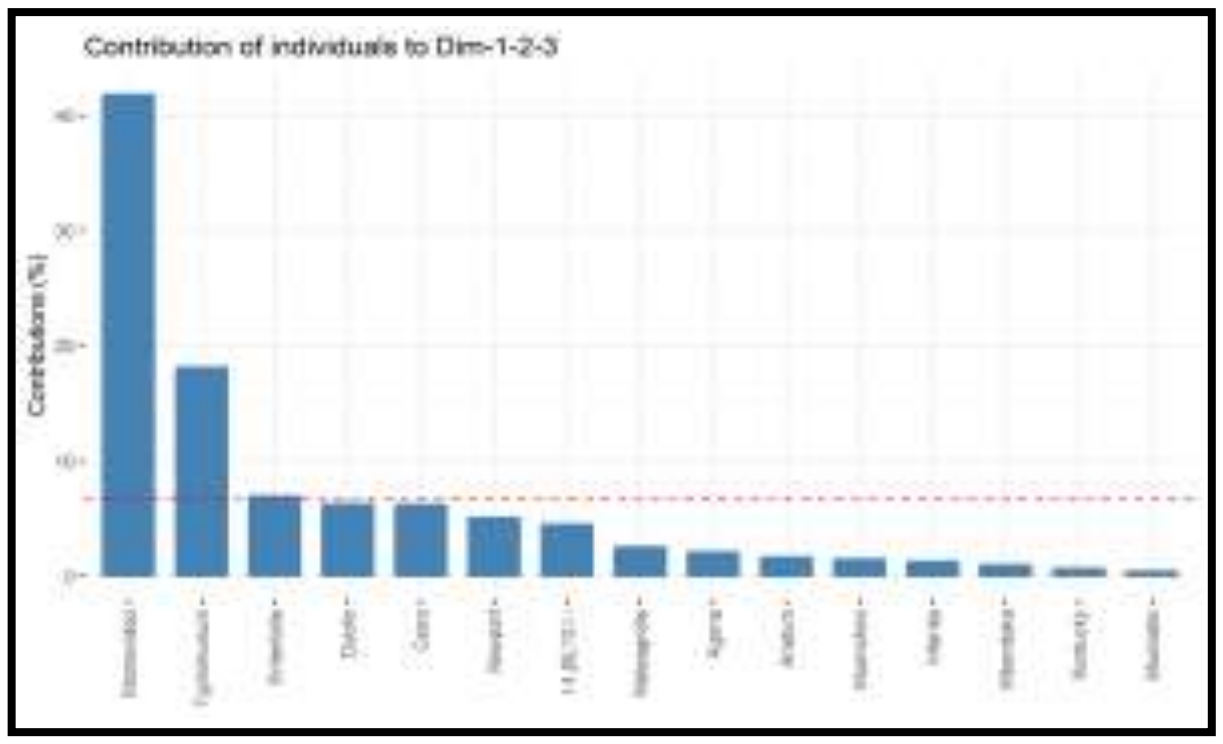

Figure S3a Contribution of individuals to Dim-1-2-3

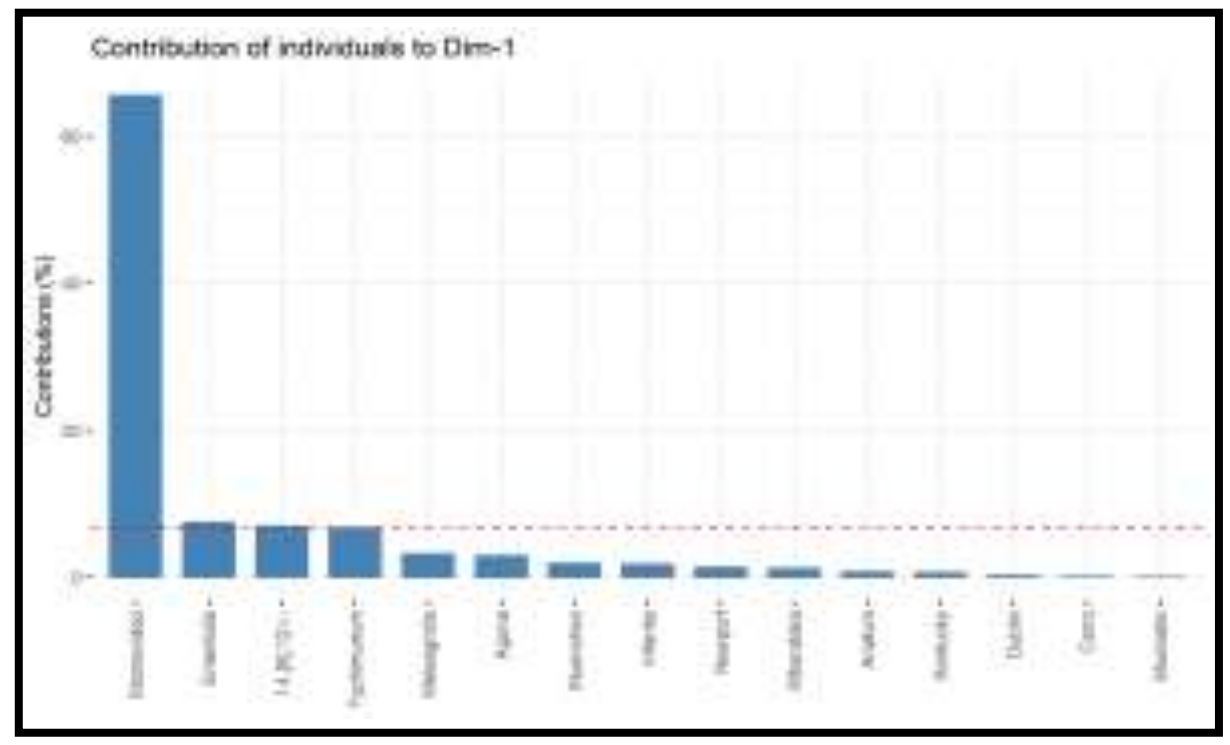

Figure S3b Contribution of individuals to Dim-1

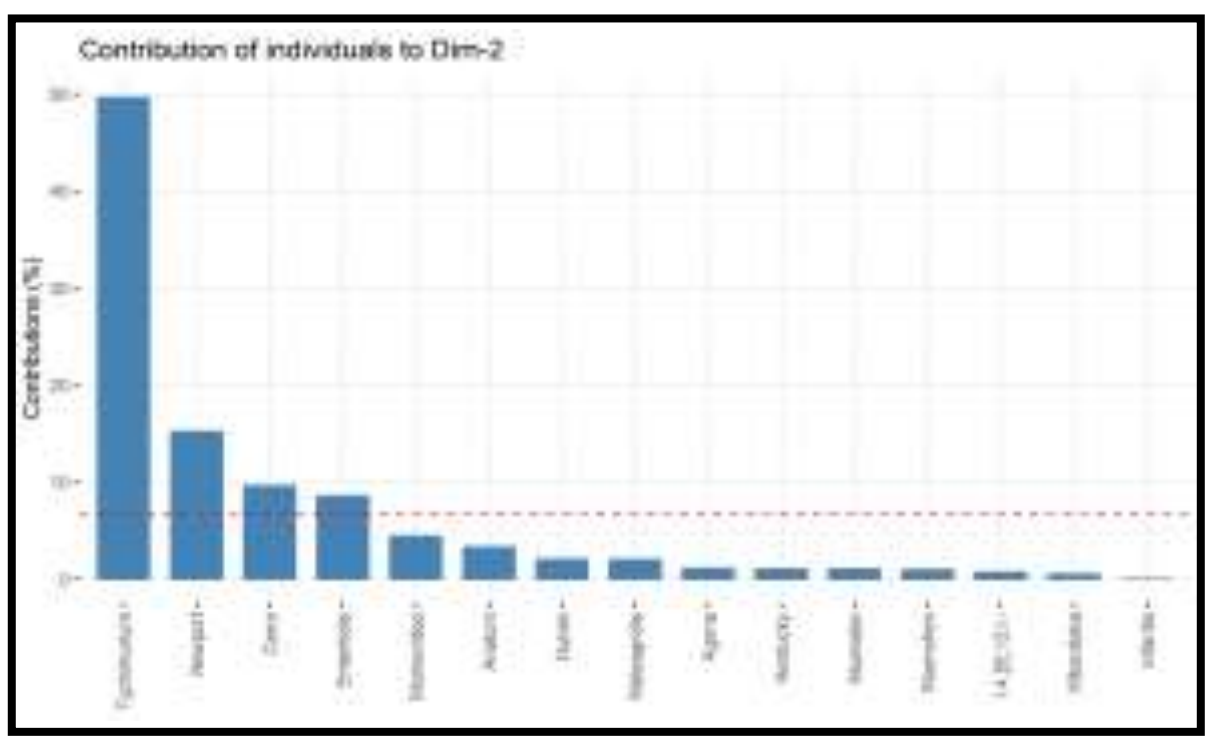

Figure S3c Contribution of individuals to Dim-2

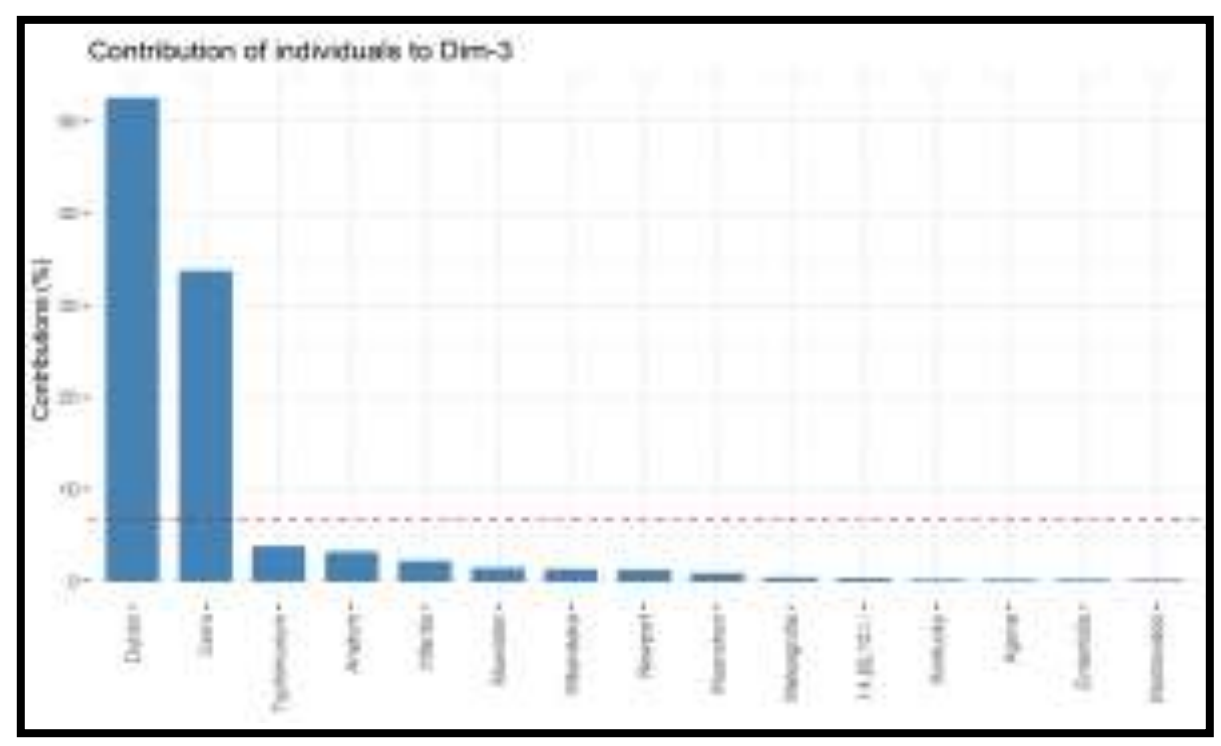

Figure S3d Contribution of individualsto Dim-3

Figure S3: Salmonella serovar contributions to principal components for different sampling sources and periods of study. Individuals measuring above the red line indicate those of which exceed the average contribution expectation.

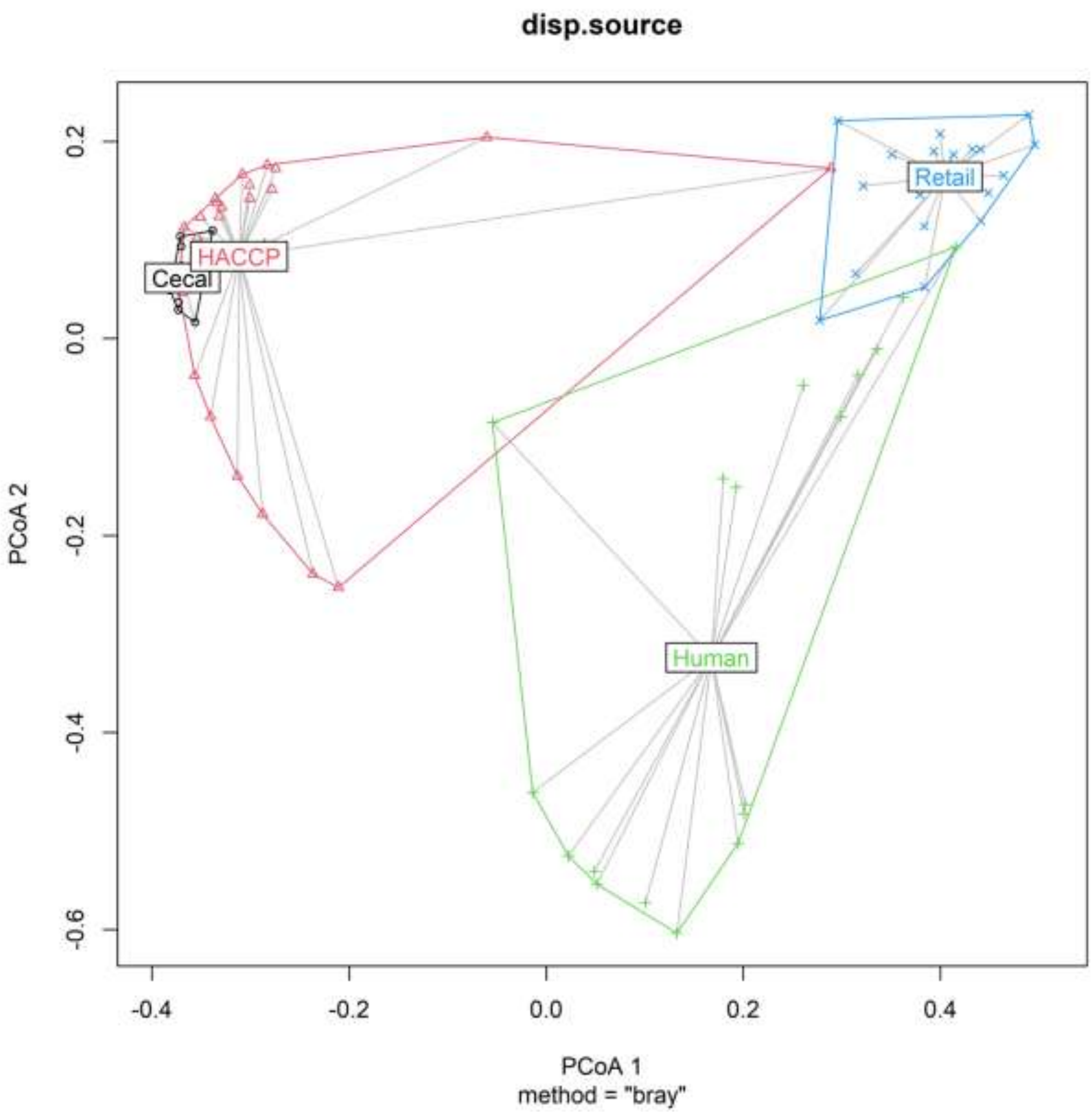

Figure S4: PCoA of sampling sources clustered based on similarity of *Salmonella* serovar composition. Data points represent each sampling source, with the distances indicating the measured dissimilarity of each sample compared to the others. Greater distance represents more variation in the composition of *Salmonella* serovars.

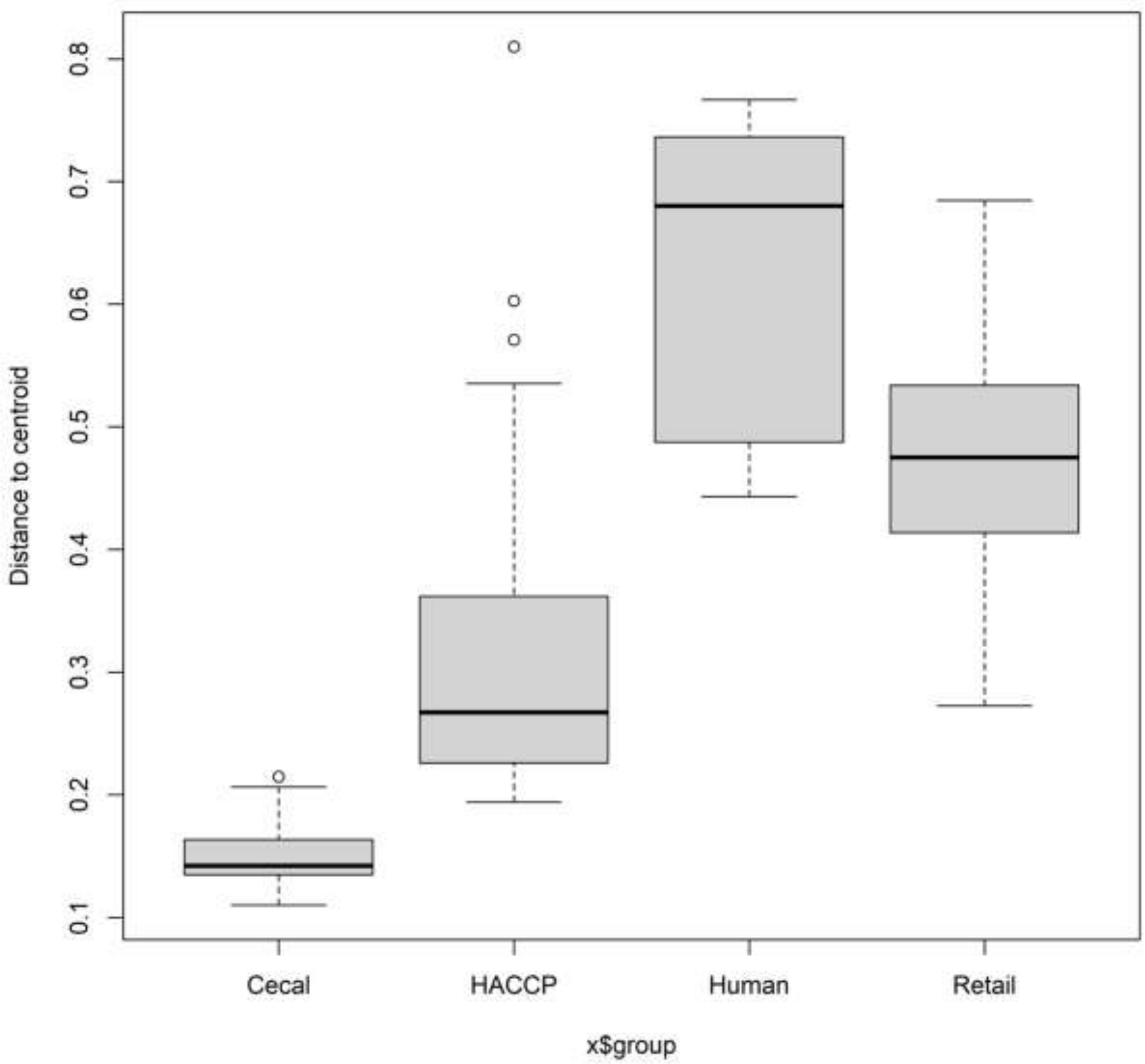

Figure S5: Distance to centroid was calculated for each sampling source to assess the variability of the composition of *Salmonella* serovars within each group. This represents the heterogeneity within each group by measuring how much each sample differs from the others within one sample source.

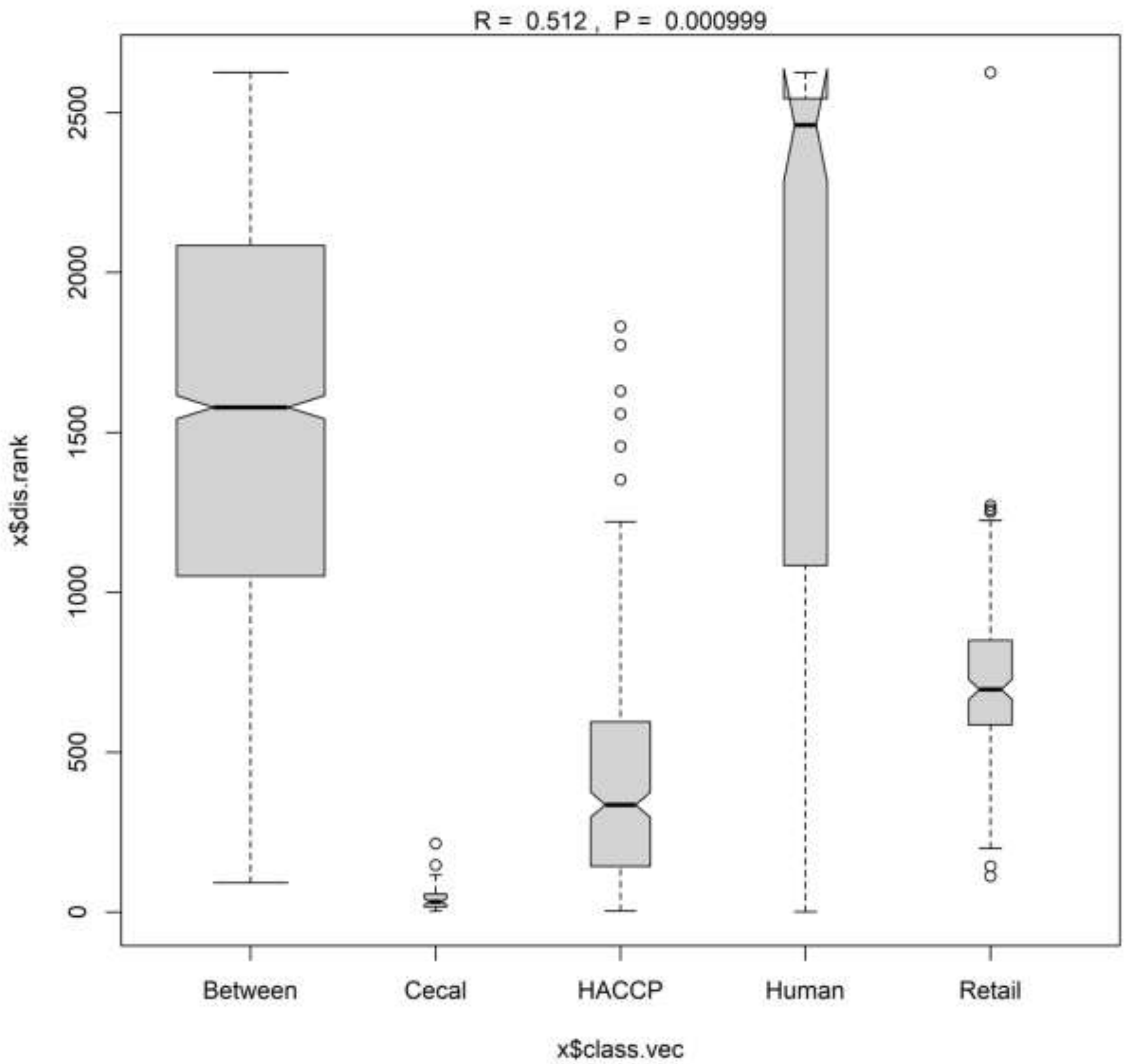

Figure S6: Analysis of similarities showing variation of *Salmonella* serovar composition across cecal, HACCP, human, and retail samples compared to the "Between" group. The bar for each sampling source represents the average (dis)similarity among each source, while the bar for the "Between" group represents the average (dis)similarity between each sampling source.

Table S3: Significant *Salmonella* serovars identified through Kruskal-Wallis test, with source comparisons, SIMPER contributions, left and right standard deviations, and left and right means.

Left_group	Right_group	SIMPER	Serovar	krusk_p	fdr_p	Left_mean	Left_sd	Right_mean	Right_sd	delta	enriched	comp_key
Cecal	Retail	0.0134	6,7:g,m,s:e,n,z15	2.00E-04	6.00E-04	0.0131	0.0129	0	0	0.0131	Cecal	Cecal vs Retail
Cecal	Retail	0.0303	Agona	1.73E-06	8.23E-06	0.0301	0.0136	0.0203	0.0507	0.0098	Cecal	Cecal vs Retail
HACCP	Retail	0.0292	Agona	2.86E-08	4.30E-07	0.0282	0.018	0.0203	0.0507	0.0079	HACCP	HACCP vs Retail
Human	Retail	0.0126	Agona	0.0049	0.0107	0.0046	0.0049	0.0203	0.0507	-0.0157	Retail	Human vs Retail
Cecal	HACCP	0.0128	Altona	2.96E-06	1.30E-05	0.013	0.0029	0.0032	0.0063	0.0098	Cecal	Cecal vs HACCP
Cecal	Retail	0.0132	Altona	5.69E-07	3.42E-06	0.013	0.0029	0.0088	0.0382	0.0042	Cecal	Cecal vs Retail
Cecal	HACCP	0.0789	Anatum	0.0133	0.0271	0.1038	0.0145	0.08	0.0485	0.0238	Cecal	Cecal vs HACCP
Cecal	Retail	0.1046	Anatum	7.33E-06	2.61E-05	0.1038	0.0145	0.0465	0.0624	0.0573	Cecal	Cecal vs Retail
HACCP	Retail	0.0773	Anatum	1.05E-08	4.30E-07	0.08	0.0485	0.0465	0.0624	0.0335	HACCP	HACCP vs Retail
Human	Retail	0.0213	Braenderup	0.0011	0.0027	0.0126	0.0148	0.0188	0.0819	-0.0062	Retail	Human vs Retail
Cecal	HACCP	0.1777	Cerro	5.09E-06	2.07E-05	0.1935	0.023	0.0459	0.0184	0.1476	Cecal	Cecal vs HACCP
Cecal	Human	0.1085	Cerro	8.46E-06	2.92E-05	0.1935	0.023	6.00E-04	8.00E-04	0.1929	Cecal	Cecal vs Human
Cecal	Retail	0.1969	Cerro	2.62E-06	1.19E-05	0.1935	0.023	0.0176	0.0407	0.1759	Cecal	Cecal vs Retail
HACCP	Human	0.0257	Cerro	2.00E-04	6.00E-04	0.0459	0.0184	6.00E-04	8.00E-04	0.0453	HACCP	HACCP vs Human
HACCP	Retail	0.0472	Cerro	3.01E-08	4.30E-07	0.0459	0.0184	0.0176	0.0407	0.0283	HACCP	HACCP vs Retail
HACCP	Retail	0.0138	Derby	1.78E-07	1.36E-06	0.014	0.013	0.0038	0.0164	0.0102	HACCP	HACCP vs Retail
Cecal	HACCP	0.0549	Dublin	3.97E-05	1.00E-04	0.0026	0.002	0.0659	0.0432	-0.0633	HACCP	Cecal vs HACCP
HACCP	Retail	0.0659	Dublin	2.74E-07	1.74E-06	0.0659	0.0432	0.1137	0.1166	-0.0478	Retail	HACCP vs Retail
Human	Retail	0.0939	Enteritidis	0.0042	0.0095	0.0886	0.085	0.0208	0.0449	0.0678	Human	Human vs Retail
Cecal	Retail	0.0146	Give	1.03E-06	5.49E-06	0.0146	0.0092	0.0081	0.0245	0.0065	Cecal	Cecal vs Retail
HACCP	Retail	0.0198	Give	9.40E-08	7.65E-07	0.0192	0.0177	0.0081	0.0245	0.0111	HACCP	HACCP vs Retail
Human	Retail	0.0137	Heidelberg	0.0049	0.0107	0.0084	0.0185	0.0123	0.0324	-0.0039	Retail	Human vs Retail
Cecal	Retail	0.0102	I 4,[5],12:i:-	6.19E-06	2.35E-05	0.0102	0.0065	0.0081	0.0353	0.0021	Cecal	Cecal vs Retail
HACCP	Retail	0.0103	I 4,[5],12:i:-	6.67E-05	2.00E-04	0.0101	0.0105	0.0081	0.0353	0.002	HACCP	HACCP vs Retail
Human	Retail	0.0624	I 4,[5],12:i:-	4.00E-04	0.001	0.0686	0.0965	0.0081	0.0353	0.0605	Human	Human vs Retail
Cecal	Retail	0.0151	Infantis	1.07E-05	3.38E-05	0.0162	0.0072	0.0537	0.0859	-0.0375	Retail	Cecal vs Retail
HACCP	Retail	0.0289	Infantis	4.36E-08	5.53E-07	0.0304	0.0168	0.0537	0.0859	-0.0233	Retail	HACCP vs Retail

Human	Retail	0.0569	Infantis	0.0201	0.0382	0.0496	0.0583	0.0537	0.0859	-0.0041	Retail	Human vs Retail
Cecal	Human	0.0233	Javiana	0.0068	0.0144	0	0	0.0245	0.0266	-0.0245	Human	Cecal vs Human
HACCP	Human	0.0241	Javiana	0.0093	0.0194	9.00E-04	0.0016	0.0245	0.0266	-0.0236	Human	HACCP vs Human
Human	Retail	0.0258	Javiana	3.00E-04	9.00E-04	0.0245	0.0266	0	0	0.0245	Human	Human vs Retail
Cecal	Retail	0.0368	Kentucky	5.81E-06	2.29E-05	0.0373	0.0099	0.038	0.078	-0.0007	Retail	Cecal vs Retail
HACCP	Retail	0.0416	Kentucky	2.13E-08	4.30E-07	0.0427	0.0152	0.038	0.078	0.0047	HACCP	HACCP vs Retail
Cecal	Retail	0.0232	Mbandaka	4.97E-05	1.00E-04	0.0239	0.0123	0.0298	0.0644	-0.0059	Retail	Cecal vs Retail
HACCP	Retail	0.0291	Mbandaka	7.15E-08	7.41E-07	0.0295	0.0152	0.0298	0.0644	-0.0003	Retail	HACCP vs Retail
Human	Retail	0.0156	Mbandaka	0.018	0.0347	0.0025	0.0029	0.0298	0.0644	-0.0273	Retail	Human vs Retail
Cecal	Human	0.0155	Meleagridis	0.0016	0.0036	0.0277	0.0148	2.00E-04	3.00E-04	0.0275	Cecal	Cecal vs Human
Cecal	Retail	0.028	Meleagridis	1.06E-06	5.49E-06	0.0277	0.0148	0.011	0.0389	0.0167	Cecal	Cecal vs Retail
HACCP	Human	0.0173	Meleagridis	2.00E-04	5.00E-04	0.0306	0.0143	2.00E-04	3.00E-04	0.0304	HACCP	HACCP vs Human
HACCP	Retail	0.0317	Meleagridis	2.18E-08	4.30E-07	0.0306	0.0143	0.011	0.0389	0.0196	HACCP	HACCP vs Retail
Cecal	Human	0.1173	Montevideo	0.0265	0.0488	0.2098	0.028	0.0725	0.229	0.1373	Cecal	Cecal vs Human
Cecal	Retail	0.2085	Montevideo	1.15E-05	3.53E-05	0.2098	0.028	0.1762	0.0976	0.0336	Cecal	Cecal vs Retail
HACCP	Retail	0.1882	Montevideo	9.17E-08	7.65E-07	0.1883	0.0693	0.1762	0.0976	0.0121	HACCP	HACCP vs Retail
Cecal	Retail	0.0296	Muenchen	3.62E-06	1.53E-05	0.0299	0.0072	0.0245	0.0444	0.0054	Cecal	Cecal vs Retail
HACCP	Retail	0.0367	Muenchen	1.69E-08	4.30E-07	0.0376	0.0298	0.0245	0.0444	0.0131	HACCP	HACCP vs Retail
Human	Retail	0.0199	Muenchen	0.0223	0.0417	0.0089	0.0107	0.0245	0.0444	-0.0156	Retail	Human vs Retail
Cecal	Human	0.0211	Muenster	0.0139	0.0278	0.0379	0.0143	0.0012	0.0016	0.0367	Cecal	Cecal vs Human
Cecal	Retail	0.037	Muenster	6.66E-06	2.45E-05	0.0379	0.0143	0.0602	0.1154	-0.0223	Retail	Cecal vs Retail
HACCP	Human	0.0303	Muenster	0.0059	0.0128	0.0541	0.0296	0.0012	0.0016	0.0529	HACCP	HACCP vs Human
HACCP	Retail	0.0524	Muenster	2.69E-08	4.30E-07	0.0541	0.0296	0.0602	0.1154	-0.0061	Retail	HACCP vs Retail
Cecal	Retail	0.0318	Newport	1.01E-05	3.38E-05	0.0337	0.0074	0.0931	0.0994	-0.0594	Retail	Cecal vs Retail
HACCP	Retail	0.0567	Newport	9.14E-08	7.65E-07	0.0563	0.0296	0.0931	0.0994	-0.0368	Retail	HACCP vs Retail
Human	Retail	0.0124	Oranienburg	0.001	0.0026	0.01	0.0145	0.0044	0.0191	0.0056	Human	Human vs Retail
Cecal	HACCP	0.0169	Reading	0.0014	0.0034	0.0025	0.0025	0.0174	0.0128	-0.0149	HACCP	Cecal vs HACCP

HACCP	Retail	0.0184	Reading	6.97E-07	3.97E-06	0.0174	0.0128	0.0102	0.0444	0.0072	HACCP	HACCP vs Retail
Human	Retail	0.0238	Saintpaul	0.0164	0.0322	0.0116	0.0122	0.0279	0.0575	-0.0163	Retail	Human vs Retail
Cecal	Retail	0.0132	Senftenberg	2.67E-07	1.74E-06	0.013	0.0042	0	0	0.013	Cecal	Cecal vs Retail
HACCP	Retail	0.0133	Senftenberg	3.02E-08	4.30E-07	0.0126	0.017	0	0	0.0126	HACCP	HACCP vs Retail
Cecal	Retail	0.0408	Typhimurium	1.06E-05	3.38E-05	0.0434	0.0144	0.1094	0.1184	-0.066	Retail	Cecal vs Retail
HACCP	Retail	0.0681	Typhimurium	5.63E-08	6.42E-07	0.0698	0.0322	0.1094	0.1184	-0.0396	Retail	HACCP vs Retail
Cecal	Retail	0.0132	Uganda	1.56E-06	7.71E-06	0.0133	0.0048	0.0073	0.0237	0.006	Cecal	Cecal vs Retail
HACCP	Retail	0.0119	Uganda	2.44E-07	1.74E-06	0.0115	0.0085	0.0073	0.0237	0.0042	HACCP	HACCP vs Retail
